# Opisthorchiasis from Imported Raw Fish

**DOI:** 10.3201/eid1012.040410

**Published:** 2004-12

**Authors:** Orit Yossepowitch, Tamar Gotesman, Mark Assous, Esther Marva, Reuven Zimlichman, Michael Dan

**Affiliations:** * E. Wolfson Hospital, Holon, Israel;; † Ministry of Health, Jerusalem, Israel

**Keywords:** Opisthrchiasis, Opisthorchis felineus, liver flukes, food-borne infection, Russia, former Soviet Union, research

## Abstract

Liver fluke infection caused by *Opisthorchiidae* is a major public health problem in many parts of the Far East, Southeast Asia, and eastern Europe. However, with the growing volume of international travel and population migration, the infection is increasingly diagnosed in countries where the disease is not endemic, particularly in North America. We report an outbreak of acute opisthorchiasis in a family that was infected in a non–disease-endemic area after eating raw carp illegally imported from a highly disease-endemic area in Siberia. With the growing numbers of former Soviet Union citizens immigrating to other countries, western physicians need to be alert regarding *Opisthorchis*-associated pathology in this population. The epidemiology and biology of *Opisthorchiidae* in the former Soviet Union are reviewed.

## Abstract 2

Acute liver fluke infection results from eating raw fish illegally imported from Siberia.

Liver fluke infection caused by trematodes belonging to the family *Opisthorchiidae*—*Opisthorchis viverrini*, *O. felineus*, and *Clonorchis sinensis*—is a major public health problem in many parts of the Far East, Southeast Asia, and eastern Europe. An estimated 17 million persons worldwide are infested: 7 million with *C. sinensis*, 9 million with *O. viverrini*, and 1.6 million with *O. felineus*. *O. viverrini* is prevalent in Thailand, Lao People's Democratic Republic, and Cambodia; *C. sinensis* is widespread in Korea, China, Taiwan, and Vietnam; and *O. felineus* is found in the Russian Federation and eastern Europe. Migration and global tourism are responsible for cases diagnosed in areas where the disease is not endemic (*1*).

The adult worms are flat, leaf-shaped, transparent, and hermaphroditic flukes that reproduce by self-fertilization. They live in the biliary and pancreatic ducts and occasionally in the gallbladder. The eggs are passed with the feces of the definitive natural host (cats, dogs, pigs, and many other fish-eating mammals) and are mature at excretion. The embryonated eggs are ingested by the intermediate host, a suitable freshwater snail, which varies geographically and according to the parasite species (*2*). In the digestive tract of the snail, the eggs hatch and become miracidia that go through several developmental stages and multiply asexually into thousands of tailed, free-swimming cercariae. The cercariae penetrate under the scales of a susceptible fish, which serves as the second intermediate host; they encyst as metacercaria, mainly in the fish body muscles. Fish belonging to the family *Ciprinidae* (carp) are the major intermediate host of *Clonorchis sinensis* and *Opisthorchis* spp (*2*). However, a wide range of species of freshwater fish can be naturally infected by liver flukes, and more than one fish species in any aquatic environment can become infected (*2*). Humans, as incidental definitive hosts, are infected by ingesting a raw fish containing metacercariae. After excysting in the duodenum, the metacercariae migrate through the ampulla of Vater into the bile ducts, where they mature into adult worms within 4 weeks and deposit yellow, operculated eggs. The parasites may live for up to 45 years in a human host, producing 1,000–2,500 eggs per day (*2*).

The infection is associated with a number of hepatobiliary diseases. The pathologic and clinical consequences of opisthorchiasis are related to the intensity and duration of cumulative infestations. The flukes cause mechanical injury to the bile ducts, and their metabolic products irritate the biliary epithelial cells, leading to cell desquamation, hyperplasia, dysplasia, and eventual fibrosis or cancer. Chronic infestation can result in obstruction of the biliary tract, dilatation of intrahepatic ducts, and subsequent cystic and saccular formations. The gallbladder may enlarge and become nonfunctional, containing muddy bile (*3*). Because adult flukes are long-lived, they can produce eggs and symptoms long after the human host has emigrated from the area (*4*). The acute symptoms of *O. felineus* infection consist of high-grade fever, malaise, anorexia, diarrhea or constipation, dull pain and discomfort in the upper right quadrant of the abdomen, arthralgia, lymphadenopathy, and urticarial skin rash. Subacute and chronic complications include suppurative cholangitis, liver abscess, and cholangiocarcinoma (*2*). Acute infestation with *C. sinensis* is usually asymptomatic, although some patients may have fever, rash, malaise, and abdominal discomfort in the right upper quadrant. Chronic clonorchiasis may be complicated with gallbladder and intrahepatic duct stones, recurrent pyogenic cholangitis, cholecystitis, liver abscess, and cholangiocarcinoma (*5*). Most persons with *O. viverrini* infection have no symptoms. Only 5%–10% of heavily infected persons have nonspecific chronic symptoms, such as right upper quadrant abdominal pain, flatulence, and fatigue. Cholangiocarcinoma is a known complication (*5*).

We report herein on a familial outbreak of liver fluke infection due to eating raw fish personally imported from Siberia. While ample information is available on the biology and epidemiology of liver fluke infection in Southeast Asia (recently summarized in a special issue of Acta Tropica [*6*]), reports in the English language literature on the situation in the former Soviet Union are scarce (*2*). Because so many persons have emigrated from the former USSR to Western countries in recent years, physicians in these countries should be more familiar with the condition; thus, review of the epidemiology of opisthorchiasis in former USSR is appropriate.

## Patients and Methods

A 46-year-old woman and her 47-year-old husband, who immigrated from Siberia to Israel 7 years earlier, were admitted because of gastrointestinal complaints of 10 days' duration. The symptoms included nausea, vomiting, yellow sclera, diffuse arthralgia (in the woman), weakness, rigors, and fever up to 39°C. On admission, the woman was afebrile, and results of her physical examination were normal. The husband's temperature was 38.4°C; his enlarged, nontender liver was palpated 3 cm below the right costal margin, and his sclera were jaundiced. Laboratory findings in both patients (Table) consisted of marked leukocytosis with notable eosinophilia and elevation of liver enzymes. Ultrasonographic examination of the abdomen showed a slightly enlarged spleen in both patients, and an enlarged liver in the husband.

**Table:** Main symptoms and laboratory findings in persons who ate raw fish imported from Siberia^a,b^

Relationship	Time	GI symptoms	Temp, °C	WBC, x10^3^ μ/L	Eosin, %	ALT/AST, U/L	ALP, U/L	LDH, U/L	Bili, mg/dL
Wife	Pretherapy	Vomiting, jaundice	38.4	58.3	78	125/96	294	528	1.69
	Posttherapy^c^	None	36.0	19.2	50	44/29	178	355	1.09
	Follow-up^d^	None	36.4	7.2	6.2	45/37	64.6	ND	0.7
Husband	Pretherapy	Nausea	39.0	16.9	50	188/164	1147	531	3.17
	Posttherapy	None	36.8	13.4	45.6	170/65	555	512	1.9
	Follow-up	None	36.6	6.85	4.5	48/35	134	390	1.17
Son	Pretherapy	None	36.5	8.2	5.7	28/31	110	368	0.77
	Posttherapy	None	ND	ND	ND.	ND	ND	ND	ND
	Follow-up	None	36.6	ND	ND	ND	ND	ND	ND
Daughter	Pretherapy	RUQ pain	36.0	7.23	4.7	33/32	53	377	0.49
	Posttherapy	None	ND	ND	ND	ND	ND	ND	ND
	Follow-up	None	36.6	ND	ND	ND	ND	ND	ND
Friend	On admission	None	36.6	8.08	1	15/14	111	372	0.64

^a^GI, gastronintestinal; Temp., temperature; WBC, leukocyte count; eosin, eosinophils; ALT/AST, alanine aminotransferase/aspartate aminotransferase; ALP, alkaline phosphatase; LDH, lactate dehydrogenase; ND, not done; RUQ, right upper quadrant. ^b^Abnormal laboratory results are underlined. Normal values: WBC (leukocyte) count, 4–10 x 10^3^ μ/L; eosinophils, 1%–6%; ALT, 0–41 U/L; AST, 0–38 U/L; ALP, 39–117 U/L; LDH, 240–480; bilirubin, 0.3–1.0 mg/dL. ^c^Posttherapy, 4 days after therapy. ^d^Follow-up, 2 weeks after therapy.

## Results

The triad of abdominal symptoms, eosinophilia, and liver enzyme impairment evoked the possibility of a helminthic infection. On further questioning, the couple recalled having eaten a smoked carp 10 days before becoming sick. The fish was bought in Nizhnevartovsk, Siberia, and was brought to Israel by the couple's son. The other members of the family, the couple's 23-year-old son and 17-year-old daughter, and a friend had also eaten the imported fish. They were asymptomatic at the time of the investigation, although the son reported a short febrile episode that resolved spontaneously, and the daughter had transient abdominal pain in the right upper quadrant. Their leukocyte counts and liver enzyme test results were normal (Table). The wife also recalled having had similar symptoms 15 years earlier, while still living in Siberia. A diagnosis of opisthorchiasis was made on the basis of ova identified in the bile from the wife.

**Figure 1:**
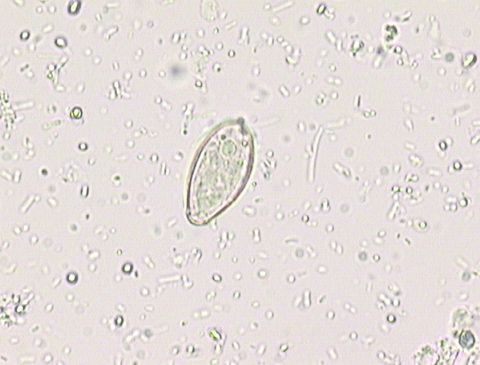
*Opisthorchis*-*Clonorchis* egg detected in the stool of one patient.

Stool samples from the five persons were examined for ova after concentration with the formaldehyde-ether technique. *Opisthorchis*/*Clonorchis* eggs (Figure 1) were found in all stool samples with the exception of that belonging to patient 5, the friend. The fish was not available for examination.

Treatment consisting of praziquantel, 25 mg/kg orally three times daily for 1 day, was administered to the infected patients (with positive stools for ova). The symptomatic patients (wife and husband) improved promptly, both clinically and as evidenced by laboratory values. On follow-up 2 weeks later, leukocyte count and liver enzyme levels had returned to normal (Table).

## Discussion

The diagnosis of liver fluke infection in this outbreak was confirmed by identifying ova in stool. Determining correctly the species of the causative parasite on the basis of egg form and shape is more challenging because eggs of *O. viverrini*, *O. felineus*, and *C. sinensis* are morphologically similar, and the differentiation is difficult even for experts (*3**,**7*). Only the identification of adult worms will confirm the species (*3*). It has been suggested that *O. felineus* differ from the other two flukes in the ratio of the length to the width of the egg, which is 1:3 in the former and 1:2 in the latter (*3*). However, we could not find additional evidence in the literature in support of this statement (*4*). Although the ova identified in our patients had a ratio of 1:2, we believe that the infection in this outbreak was caused by *O. felineus* on the basis of the source of the consumed fish and the acute symptoms in two of the four infested family members. In fact, Nizhnevartovsk is located in the Ob River basin, where *O. felineus* infection is hyperendemic (*8*). In addition, acute serum sickness–like symptoms are much more common after *O. felineus* infection than after infection with *O. viverrini* or *C. sinensis* (*4**,**5*). Moreover, *O. viverrini* is not endemic in the Russian Republic, while *C. sinensis* is found only in the Amur River area on the Russian-Chinese border (*2*).

**Figure 2:**
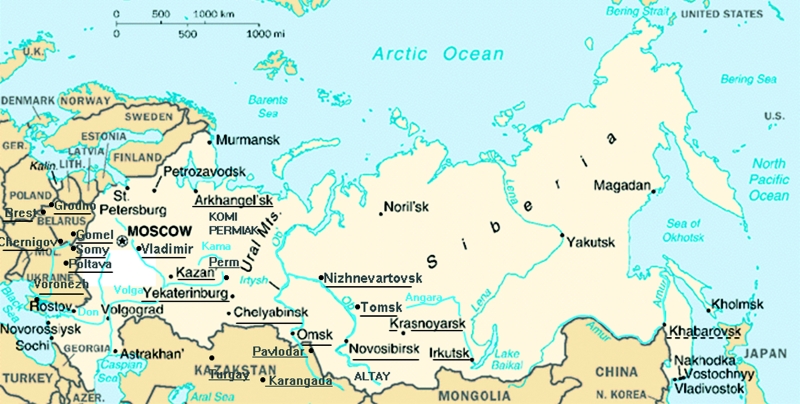
*Opisthorchis* (solid lines) and *Clonorchis* (broken lines) endemic areas in the former USSR. Original map was obtained from the United Nations Development Programme Web site (www.undp.org).

*O. felineus* infection is the most prevalent foodborne liver fluke infection of humans in Russia, Ukraine, and Kazakstan (Figure 2). Infestation usually follows consumption of raw, slightly salted, and frozen fish ("stroganina"). The parasite is endemic in an area that covers nearly all the territory of the Russian Federation with the exception of the northern parts of Siberia and the far-eastern regions. The largest parasite-endemic area is in western Siberia, namely the Ob and Irtysh River valleys and their tributaries (*9**–**12*). In the central part of this area, the Tyumen and Tomsk Districts, the mean prevalence of human infection is 40%–95%. Prevalences of 45% to 65% were reported in the Komi-Permiak national district, and infection rates up to 46% have been documented in some communities in Omsk District. Other districts and territories where opisthorchiasis is endemic include the Yekaterinburg (formally Sverdlovsk) District (*13*), Altai territory, Voronezh District (*14*), Volga River valley (*15*) and Archangelsk District in western Russia, and the Angara River (*16*), Krasnoyarsk territory, and Irkutsk District in eastern Siberia (*2*). In Ukraine, opisthorchiasis is limited to the Sumy, Poltava, and Chernigov Districts of the Dnieper River basin (*17**,**18*), where the prevalence is 5%–40%. In Kazakstan, opistorchiasis is endemic in the Aktyubinsk, Dzhezkazgan, Karaganda, Pavlodar, Tselinograd, and Turgay Districts. Foci of opisthorchiasis have also been found in the Brest, Gomel, and Grodno provinces of Belarus (*2*). Limited endemic foci of opisthorchiasis in some areas of the Baltic States, eastern Germany, and Poland were described before the Second World War; however, no recent information on the occurrence of the infection in humans in these countries is available.

The correlation between *O. felineus* infection and cholangiocarcinoma was studied in the Tyumen region, Russia. In the southern part of the region, where 0.5% of the population was infected with *O. felineus*, the prevalence of cholangiocarcinoma was 4.4 per 100,000 population. In the central area of Tyumen with 45% prevalence of *O. felineus* infestation, the rate of cholangiocarcinoma was 10-fold higher than in the south (49.8 per 100,000 population) (*2*).

*C. sinensis* infection is endemic in the Amur River valleys and Khabarovsk territory, situated in the far eastern part of the Russian Federation. The prevalence of the infection in the native Nanai population is 24% in the most affected villages (*2**,**19*).

The major snail hosts for *O. felineus* are *Codiella (Bithynia) inflata*, *C. troscheli*, and *C. leachi*. *C. sinensis* is transmitted by a wide range of operculate snails, *Parafossarulus manchouricus* being the main one (*2*). Twenty-two species of 17 genera of the family *Ciprinidae* are infected by *O. felineus*; the most important are *Leuciscus idus*, *L. leuciscus*, and *Rutilus rutilus*; five species host the *C. sinensis* fluke (*2*).

The growing volume of international travel and population migration, facilitated by increasing availability of air transportation, is responsible for cases of opisthorchiasis and clonorchiasis diagnosed in non–disease-endemic countries, mainly in North America. Most reports from the United States and Canada describe the detection of *Opisthorchis*/*Clonorchis* ova in immigrants from Southeast Asia with chronic infection (*20**–**27*). The infection was also documented in North American residents who contracted the disease during long-term or short visits to disease-endemic areas (*22*). In the United States, liver fluke infection continues to be an active health problem for hundreds of thousands of Southeast Asian refugees who have immigrated since 1975. *Clonorchis* infestation was documented in 26% of 150 Chinese immigrants in New York City (*21*). Stool examinations of 186 Indochinese refugees in California have detected *C. sinesis* eggs in 13% (*23*). In another report, the prevalence of *Opisthorchis* eggs among 226 asymptomatic adult Southeast Asian immigrants to the United States was 11% (24). In Montreal, Canada, *Clonorchis* infestation was documented in 15.5% of 400 Chinese immigrants (*20*).

We have identified only two reports (in German) of patients of Russian origin whose conditions were diagnosed in western Europe. A 58-year-old woman, who emigrated from Tomsk a year earlier, was seen in Wiesbaden, Germany, for right upper abdominal and flank pain, reduced appetite, and weight loss; *O. felineus* eggs were detected in stool and duodenal aspirate (*28*). For two patients from Siberia with suspected eosinophilic leukemia and carcinoma of the gallbladder, respectively, opisthorchiasis was diagnosed in Hamburg (*29*).

Unlike previous reports of opisthorchiasis diagnosed in non–disease-endemic countries, which included patients infected in areas endemic for disease, the patients in the present series were infected outside an endemic region by food imported illegally from a country where the disease was highly prevalent. An estimated 2 million citizens from the former USSR have moved since the Soviet collapse in 1989, mostly to North America, western Europe (mainly Germany), and Israel (*30*). Most of these immigrants continue to maintain strong cultural ties with their countries of origin, including through eating delicatessen food from the "old country." Thus, both chronic and acute infections can be diagnosed in this population. Physicians providing care to immigrants from the former Soviet Union should be aware of the potential presence of liver fluke infection in these patients and consider the entity in the differential diagnosis, when appropriate.
